# Editorial: Next generation sequencing (NGS) for rare diseases diagnosis ‐ Volume II

**DOI:** 10.3389/fgene.2023.1249585

**Published:** 2023-07-10

**Authors:** Xiu-An Yang, Hu Hao, Can Liao

**Affiliations:** ^1^ Laboratory of Genetic Engineering and Genomics, School of Basic Medical Sciences, Chengde Medical University, Chengde, China; ^2^ Hebei Key Laboratory of Nerve Injury and Repair, Chengde Medical University, Chengde, China; ^3^ Department of Pediatrics, The Sixth Affiliated Hospital, Sun Yat-sen University, Guangzhou, China; ^4^ Inborn Errors of Metabolism Laboratory, The Sixth Affiliated Hospital, Sun Yat-sen University, Guangzhou, China; ^5^ Guangzhou Women and Children’s Medical Center, Guangzhou, China

**Keywords:** next generation sequencing, rare diseases diagnosis, whole-exome sequencing, whole-genome sequencing, copy number variants (CNV) sequencing

## Introduction

Next generation sequencing (NGS) has greatly facilitated the diagnosis of rare diseases. According to a 2013 statistic, 87% of Mendel’s genetic disease-causing genes were discovered by NGS technology from 2007 to 2013 (McKusick, 2007). To emphasize the significance of NGS and present high-quality research on prevention, diagnostics, and treatment, we previously launched a Research Topic in Frontiers in Genetics. Due to popular demand, the Research Topics were extended as Volume II. A totally of 16 articles were published in this volume, comprising 16 articles covering various topics such as preimplantation and prenatal diagnosis, case reports of rare diseases, multiple case summaries, and hematological tumors.

## Preimplantation and prenatal diagnosis

Giving birth to a healthy baby (or babies) is an exceptional gift for families with genetic diseases. In the case of a male proband from unrelated healthy parents, several complications arose during the first pregnancy, including extensive neurogenic damage, developmental delay, and an enlarged heart (Shi and Ye). To determine the genetic pathogenic variant, karyotype analysis, Fluorescence *in situ* hybridization (FISH), whole exome sequencing (WES), and copy number variants (CNV) sequencing were conducted. WES revealed heterozygous variants in *DUOX2* (c.1588 A >T (p.K530X), c.2654G >T (p.R885 L)), respectively inherited from the mother and father. Although the karyotype analysis was normal, CNV sequencing showed a *de novo* Xq28-q28 duplication (5.59 Mb × 2) and 18q22.2-q23 deletion (9.85 Mb × 1) in the proband. FISH revealed that the mother was a carrier of a reciprocal translocation (RecT) between ChrX and Chr18 [t (X; 18) (q28; q22.2)]. To identify embryos free of pathogenic variants, preimplantation genetic testing was conducted. This was followed by amniocentesis at gestational week 20, which confirmed the fetal genetic makeup. Ultimately, a healthy infant was delivered. This case report underscores the importance of using NGS in preimplantation prenatal diagnosis. Furthermore, the study highlights the need for a multi-technical approach that includes karyotype analysis, FISH, and linkage analysis in reproductive medicine.

Yu et al. reported a case of fetal tetrasomy 9p, which was initially identified by non-invasive prenatal testing (NIPT). The 37-year-old pregnant woman was healthy and had undergone NIPT due to advanced maternal age. At 12 weeks of gestation, an ultrasound screening revealed normal nuchal translucency (NT) and nasal bone. Unfortunately, at 15 weeks of gestation, the NIPT indicated a duplication at 9p24.3p11.2. The result was validated through amniocentesis, karyotyping, chromosome microarray (CMA), and FISH. Based on the opinions of the pregnant woman and her husband, the pregnancy was terminated. It is recommended that NIPT should be carried out in cases of advanced maternal age to rule out fetal genetic variations, even in the absence of fetal abnormalities revealed by ultrasound examination.

Norrie disease (ND) is an X-linked recessive disorder that can result in blindness in early postnatal stages due to retinal detachment (Meire et al., 1998). A 29-year-old pregnant woman gave birth to a son who was born with congenital bilateral blindness, but showed no signs of cognitive impairment or deafness (Li et al.). Prenatal diagnosis was conducted at 16 weeks of gestation, and WES identified a hemizygous variant, c.174 + 1G > A, in the *NDP* splicing site. *In vitro* splicing assays were performed on mRNA using HeLa and 293 T cells to study the effects of the *NDP* c.174 + 1G > A variant on splicing. The study highlighted the essentiality of NGS combined with relevant laboratory tests for diagnosing genetic diseases.

Short-rib thoracic dysplasia (SRTD), with or without polydactyly, is a group of autosomal recessive, genetically heterogeneous skeletal dysplasias. Mutations in the *DYNC2H1* gene have been identified as the genetic cause of short-rib thoracic dysplasia 3 with or without polydactyly (SRTD3; OMIM #613091) (Bonafe et al., 2015). The study conducted by Chen et al. included unrelated cases visiting for pregnancy check-up, both of whom had a history of pregnancy termination due to fetal dysplasia. WES identified two compound heterozygous variations in the *DYNC2H1* gene for one patient, namely c.2386C >T (p.Arg796Trp) and c.7289 T >C (p.Ile2430Thr), and exon (64–83) deletion and c.8190G >T (p.Leu2730Phe) for the other patient. Sanger sequencing validated the results, and RT-PCR analyses confirmed that the variants were inherited from the parents, who were carriers of the mutations. Chen et al. provided an extensive description of the pathogenic genes and *DYNC2H1* variants previously identified in SRTD.

The Ehlers-Danlos syndromes (EDS) are a group of connective tissue diseases that can be inherited. A specific subtype of EDS, known as Musculocontractural Ehlers-Danlos syndrome (mcEDS), can be caused by mutations in the carbohydrate sulfotransferase 14 (*CHST14*) or dermatan sulfate epimerase genes (Malfait et al., 2010). In this case, a 34-year-old woman who was 22 weeks pregnant had an ultrasound that showed her fetus had adduction flexion in the feet (Zhou et al.). She then underwent prenatal testing, which included WES, CNV sequencing, and amniotic fluid cell karyotype analysis. Two potential genetic causes for mcEDS were identified as variants in the *CHST14* gene (NM_130468.3 c.958C >T (p.Arg320*) and NM_130468.3 c.896A>G (p.Tyr299Cys)). This study has expanded our understanding of the genetic causes of mcEDS.

## Case reports of rare diseases

Before the advent of NGS, due to limitations in research methods, many pathogenic genes and sites of rare diseases were not fully studied. This resulted in a number of extremely rare diseases with only dozens or even fewer cases reported worldwide. In this volume, three extremely rare case reports were published. One case involved a healthy, non-consanguineous Chinese couple who gave birth to a full-term infant girl (Chen et al.). Unfortunately, the baby died at a few months of age due to severe respiratory infection and unknown respiratory failure. WES identified novel compound heterozygous splicing mutations in *RFX5*, namely c.353 + 6T >G and c.757 + 1 G >A. The results were confirmed through Sanger sequencing, and the pathogenicity of RFX5: c.353 + 6T >G was further investigated using RT-PCR. RFX5 is a protein with two DNA-binding domains (DBDs), and the c.353 + 6T >G splicing mutation could lead to a truncated DBD. This could impair the ability of RFX5 to bind to the MHC II promoter and lead to reduced transcription of MHC II molecules. This study thus contributes to the extension of the genetic spectrum of MHC class II deficiency.

Biallelic variants in the *TENM3* gene can result in two distinct phenotypes: nonsyndromic microphthalmia with coloboma-9 (MCOPCB9) and microphthalmia and/or coloboma with developmental delay (MCOPS15) (Aldahmesh et al., 2012; Chassaing et al., 2016). To date, only eight cases have been documented. Lu et al. recently reported two cases of syndromic microphthalmia associated with *TENM3* variants . In one case, a 5-month-old girl was found to have compound heterozygous variants (p.Leu1283_Ser1285del; p. Thr1233Thrfs*20) in *TENM3* through trio-based WES. The p. Leu1283_Ser1285del variant was inherited from her mother, while TA cloning indicated that the other was a *de novo* mutation located on the Father-derived chromosome. The second patient was found to have compound heterozygous variants in the *TENM3* gene: c.6464T > C; p. Leu2155Pro and c.941C > T; p. Ala314Val, respectively inherited from each parent. The authors provided a clear description of the clinical presentation and summarized the features of all reported cases with *TENM3* variants to date.

The Cdc42 molecule is a small GTPase that belongs to the Rho GTPase family and plays multiple important physiological functions in regulating the cell cycle. The heterozygous c.191A >G (p.Tyr64Cys) variant is reported as the pathogenic molecular etiology of Takenouchi-Kosaki syndrome (TKS). TKS is a range of phenotypic syndromes characterized by psychomotor development, dysmorphic facial features, and hematologic or lymphatic defects (Martinelli et al., 2018). In a male infant, a genetic syndrome was suspected due to facial dysmorphism, low-set ears, axial hypotonia with peripheral spasticity, camptodactyly, and cutaneous syndactyly of toes (Szczawińska-Popłonyk A et al.). WES revealed the c.191A >G; p. Tyr64Cys (NM_001791.4) variant in the *CDC42* gene, which was later confirmed through Sanger sequencing. While this study did not identify novel genetic sites, it does expand the clinical spectrum of TKS.

## Multiple case summaries

With the wide application of NGS, an increasing number of relationships between pathogenic genes and the phenotypes of monogenic genetic diseases have been established. Additionally, the mutation sites of Mendelian disease-causing genes have been widely reported. It is well-documented that mutations in the *ATP7B* gene could lead to an autosomal recessive disease called Wilson’s disease (WD). In this volume, 39 and 30 genetically diagnosed WD cases were respectively studied by Wang et al. and Zhou et al.The participants in Wang’s study were from Yunnan province in China, including seven different ethnic groups. Homozygous mutation frequency is higher, whereas protein-truncating variant (PTV) is less frequent in ethnic minorities compared with the Han population found that the frequency of p. R778L and p. I1148T is relatively high. They suggested that elevated alanine transaminase (ALT), decreased ceruloplasmin level, and increased 24-h urinary copper level should be considered as three important indices for rapid diagnosis.

Autism spectrum disorder (ASD) is a cluster of common neurological developmental symptoms in the pediatric population. In this study, Zhang et al. recruited 354 ASD candidates and investigated the genetic etiology using WGS and RNA sequencing Pathogenic variants including single nucleotide variations (SNV), CNV, and structural variations (SV) were investigated. They found that ASD affected children had a relatively low frequency of rare CNV and SNV variants. The total positive rate (5.3%) was relatively lower than in other studies, and the authors attributed this to the mutation characteristics they focused on. It is worth noting that the authors used RNA sequencing and RT-PCR to validate the variants. Therefore, the false positive rate of mutations will be reduced.

Variants in the *LMNA* gene can give rise to a range of clinical manifestations called laminopathies. To explore the genetic basis of LMNA-related muscular dystrophies, Cesar et al. enrolled 26 patients from different parts of the world and sequenced 105 genes using a targeted approach. They identified that the most severe phenotypes resulted from the coexistence of a rare damaging variant in *LMNA* and another damaging variant in a gene involved in NMD. The authors highlighted the marked genetic heterogeneity of laminopathies and advocated for comprehensive genetic testing of both LMNA and other genes linked to muscle disorders to reveal potential causal mechanisms.

Fabry disease (FD) arises from variants in the X chromosome-located *GLA* gene, which encodes alpha-galactosidase A (α-Gal A). Abnormal expression or function in α-Gal A activity can cause the buildup of trihexosaccharide sphingolipid alcohol (GL-3) and its derivative, deacetyl GL-3 (Lyso-GL-3). The clinical manifestations of FD are highly heterogeneous. Li et al. retrospectively analyzed ten patients who visited their hospital and were diagnosed with FD. They provided a detailed description of the patients’ clinical manifestations, laboratory and auxiliary examinations, FD-specific indexes and GLA genetic testing, pedigree screening, and treatment. The authors emphasized that it is critical for doctors to understand the clinical symptoms of FD and actively screen for early detection and diagnosis of the disease.

*CTNS* encodes a lysosomal cystine transporter cystinosin, and variants in this gene can cause the rare hereditary autosomal recessive disorder known as nephropathic cystinosis. Disruption of cystinosin function can lead to cystine accumulation in cells, particularly in the kidneys. Cystinosis can be classified into three subtypes based on symptom severity and age of onset. In a study conducted by Savostyanov et al. 40 children who were clinically diagnosed with nephropathic cystinosis and confirmed by molecular testing were examined. The methods employed included tandem mass spectrometry, Sanger sequencing, multiplex PCR, MLPA, and haplotype analysis. The researchers confirmed that the most common pathogenic variant was a 57 kb deletion, 22.5% of alleles were novel, and there was a founder effect for the c.1015G > A and c.518A > G variants in the Karachay and Chechen ethnic groups, respectively.

Café-au-lait macules (CALMs) are common birthmarks that can increase in number and range over time. They are found in 2%–3% of healthy newborns, and the occurrence of six or more typical CALMs indicates neurofibromatosis type 1 (NF1), which is caused by variants in the *NF1* gene. In a study by Zhong et al. the variant characteristics in six Chinese Han populations diagnosed with CALMs were investigated. Sequencing revealed two novel variants in NF1—NC_ 000017.11(NM_001042492.2):c.7355G >A and NC_000017.11(NM_001042492.2):c.2739_2740del. The authors also used dermoscopy and reflectance confocal microscopy to demonstrate the features of CALMs. The skin imaging characteristics provided by the authors can assist clinicians in making decisions during CALMs diagnosis.

## Hematological tumor

The incidence of tumors in children is generally low. However, acute lymphoblastic leukemia (ALL) is often observed in this population, which is highly correlated with altered lymphoid precursor hyperplasia. Furthermore, *IKZF1* variants are frequently observed in children diagnosed with ALL. Zhang et al. conducted a study analyzing the clinical and genetic features of 200B-cell ALL pediatric patients by utilizing MLPA and targeted NGS methodologies. Their results indicate that *IKZF1* variants were identified in 22 patients and there was a significant association between *IKZF1* variants and higher WBC counts. Moreover, the study revealed that patients with *IKZF1* mutations had decreased sensitivity to glucocorticoid induction and higher levels of minimal residual disease compared with patients with *IKZF1* wildtype. This research sheds light on the association between genetic mutations and clinical features, providing new perspectives for targeted therapy in ALL.
